# Exendin-4 reduces food intake via the PI3K/AKT signaling pathway in the hypothalamus

**DOI:** 10.1038/s41598-017-06951-0

**Published:** 2017-07-31

**Authors:** Yan Yang, Pique P. Choi, Wanli W. Smith, Weijie Xu, Delin Ma, Zachary A. Cordner, Nu-Chu Liang, Timothy H. Moran

**Affiliations:** 1 Department of Endocrinology, Tongji Hospital, Tongji Medical College, Huazhong University of Science and Technology, Wuhan, Hubei China; 2 Department of Psychiatry and Behavioral Sciences, Johns Hopkins University School of Medicine, Baltimore, Maryland United States of America; 3 Department of Pharmaceutical Sciences, University of Maryland School of Pharmacy, Baltimore, Maryland United States of America; 4 Department of Psychology, University of Illinois-Urbana Champaign, Champaign, Illinois United States of America; 5 Johns Hopkins Global Obesity Prevention Center, Johns Hopkins University, Baltimore, Maryland United States of America

## Abstract

Exendin-4 (EX-4), a glucagon-like peptide-1 (GLP-1) receptor agonist, has been shown to reduce food intake and to increase proopiomelanocortin (*POMC*) gene expression in the hypothalamus. In this study, we examined the potential neural mechanisms by which these effects occur. Male Sprague Dawley rats were implanted with a cannula in the third ventricle of the brain through which an inhibitor of phosphatidylinositol-3 kinase (PI3K) (wortmannin) was administered, and EX-4 or vehicle was administered via intraperitoneal (IP) injection. The activity of PI3K/protein kinase B (AKT) and insulin receptor substrate-1 (IRS-1) in the hypothalamic arcuate was determined. We found that EX-4 treatment significantly decreased food intake and body weight. However, there were almost no changes in food intake and body weight when wortmannin injection (into the third ventricle) occurred prior to EX-4 IP injection. EX-4 not only increased the activity of PI3K/AKT, but it also increased IRS-1 activity. These results show that EX-4 likely suppresses food intake due to its ability to enhance insulin signaling.

## Introduction

The glucagon-like peptide-1 (GLP-1) system was recently established as a target for type 2 diabetes (T2D) and obesity treatment^1–3^, as it improves blood glucose regulation, reduces food intake, and helps to manage body weight^4, 5^. Human and nonhuman animal experiments have shown that GLP-1 affects the central nervous system (CNS) to alter food intake and body weight regulation. In human studies, the GLP-1 receptor (GLP-1R) ligand greatly enhances insulin release^6^ and reduces appetite and energy intake^7^, when it is peripherally administrated. In animal experiments, either peripheral or central administration of GLP-1R suppressed food intake at least in part via CNS GLP-1R activation^8–10^. In addition, centrally injected GLP-1R antagonist exendin-(9–39) can attenuate the intake suppression effects of the GLP-1R agonist^11, 12^; centrally, viral knockdown of endogenous CNS GLP-1-producing preproglucagon (PPG) neurons causes hyperphagia and increased body weight^13^. Recently, a study by Rupprecht *et al*.^14^ showed that GLP-1R activation in the hindbrain suppressed food intake via a phosphatidylinositol-3 kinase (PI3K)/protein kinase B (AKT)-dependent pathway, which started to elucidate the specific CNS GLP-1R-expressing nuclei and the intracellular signaling mechanisms through which food intake suppression occurs. However, additional studies are needed to identify the role(s) of CNS GLP-1R in food intake and energy expenditure.

Food intake and energy expenditure is mediated in part by neuropeptides expressed in neurons within nuclei of the mediobasal hypothalamus^15, 16^. Orexigenic neuropeptides, such as neuropeptide Y (*NPY*) and agouti-related protein (*AgRP*), increase food intake and body weight, while α-melanocyte-stimulating hormone (*α-MSH*), a product of proopiomelanocortin (*POMC*), is an anorexigneic neuropeptide that reduces food intake. *α-MSH* is expressed in neurons of the hypothalamic arcuate nucleus (ARC), which are perfectly positioned to integrate short-term satiety and long-term adiposity signals for energy homeostasis^16, 17^. PI3K/AKT signaling is thought to mediate leptin and insulin action in *POMC* and* AgRP*-expressing neurons^18^. It has been suggested that insulin helps to suppress the urge for food intake in the CNS via PI3K/AKT signaling^19^. Upstream to PI3K/AKT in the insulin pathway, the activities of the β-subunit of the insulin receptor and insulin receptor substrate-1 (IRS-1)^20, 21^ are elevated in response to the ratio of phospho-insulin receptor/total insulin receptor and phospho-IRS-1/total IRS-1.

Our previous study suggested that GLP-1R agonist exendin-4 (EX-4) reduced food intake and body weight by altering *POMC* gene expression in the hypothalamus of rats^8^. However, whether insulin signaling is involved in the mechanism by which EX-4 reduces food intake remains unknown. CNS GLP-1R signaling is involved in energy balance^22^, while PI3K/AKT signaling is also required in the regulation of energy expenditures within the hypothalamus^23^. In addition, PI3K/AKT signaling is both the direct and indirect upstream target of *POMC* gene expression^24–26^. These data suggest that the intake suppressive effects of GLP-1R require PI3K/AKT signaling regulation.

To test our hypothesis, intracerebroventricular (ICV) administration of a PI3K inhibitor via the third cerebral ventricle and intraperitoneal (IP) injection of EX-4 was used to explore the mechanisms by which peripheral administration of EX-4 reduces food intake in the hypothalamic ARC *in vivo*. We found that the effects of ICV wortmannin on EX-4-induced reduced food intake occurred by increasing ARC PI3K signaling.

## Results

### Low dose ICV wortmannin did not affect food intake

**Figure 1:**
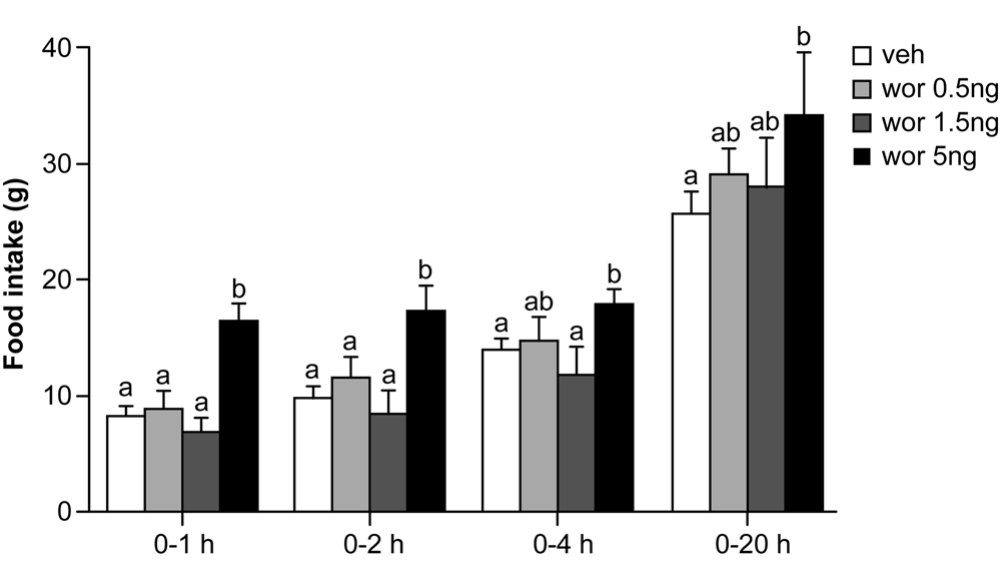
Food intake at 1, 2, 4, and 20 h after third ICV administration of different doses of wortmannin or vehicle. Food intake did not significantly increase in response to 0.5 ng or 1.5 ng wortmannin, but increased in response to 5 ng wortmannin. Data are represented as mean ± SEM (N = 4 for each group). In each panel, groups that share the same letter (eg. “a” and “ab”) are not significantly different from each other after obtaining a significant main effect by two-way ANOVA.

To identify the highest dose of wortmannin that inhibits PI3K in ARC without increasing food intake, we first conducted a dose-response test. Two-way ANOVA, Dose × Time, revealed a main effect of Dose [F(1,52) = 23.65, *P* *<* 0.001] and Time [F(3, 52) = 18.6, *P* *<* 0.001]. There was no significant Dose × Time effect [F(9, 52) = 0.93, *P* *=* 0.509]. The results are summarized in Fig. 1.

Administration of vehicle or 0.5 ng wortmannin resulted in similar food intake at every time point measured: 0–1 h (vehicle *vs*. 0.5 ng: 8.8 ± 0.6 g *vs*.9.3 ± 1.0 g, post-hoc *P* *=* 0.70), 0–2 h (vehicle *vs*. 0.5 ng: 9.8 ± 0.6 g *vs*.11.0 ± 1.1 g, post-hoc *P* *=* 0.37), 0–4 h (vehicle *vs*. 0.5 ng: 13.9 ± 0.4 g *vs*.14.5 ± 1.3 g, post-hoc *P* *=* 0.68) and 0–20 h (vehicle *vs*. 0.5 ng: 24.9 ± 0.8 g *vs*. 28.1 ± 1.4 g, post-hoc *P* *=* 0.24). While administration of 1.5 ng wortmannin appeared to decrease food intake at 0–1, 0–2, and 0–4 h and to increase food intake in 0–20 h, food intake was not significantly different compared to vehicle treatment [vehicle *vs*.1.5 ng: 0–1 h, 8.8 ± 0.6 g *vs*.6.7 ± 1.2 g, post-hoc *P* *=* 0.12; 0–2 h, 9.8 ± 0.6 g *vs*.7.7 ± 1.1 g, post-hoc *P* *=* 0.13; 0–4 h, 13.9 ± 0.4 g *vs*.11.0 ± 1.5 g, post-hoc *P* *=* 0.07; 0–20 h, 24.9 ± 0.8 g *vs*. 27.3 ± 1.7 g, post-hoc *P* *=* 0.38]. Finally, ICV administration of 5 ng wortmannin significantly increased food intake at 0–1 h (vehicle *vs*.5 ng: 8.8 ± 0.6 g *vs*.15.8 ± 0.7 g, post-hoc *P* *<* 0.001), 0–2 h (vehicle *vs*.5 ng: 9.8 ± 0.6 g *vs*.16.6 ± 0.7 g, *P* *<* 0.001), 0–4 h (vehicle *vs*.5 ng: 13.9 ± 0.4 g *vs*. 17.4 ± 0.4 g, *P* *<* 0.05), and 0–20 h (vehicle *vs*.5 ng: 24.9 ± 0.8 g *vs*.34.2 ± 2.9 g, *P* *<* 0.005) after a 4-hour fasting period.

In summary, 0.5 ng and 1.5 ng wortmannin injection did not significantly affect food intake, but 5ng wortmannin injection did.

### Wortmannin reversed the suppression of food intake caused by intraperitoneal EX-4 injection

**Figure 2:**
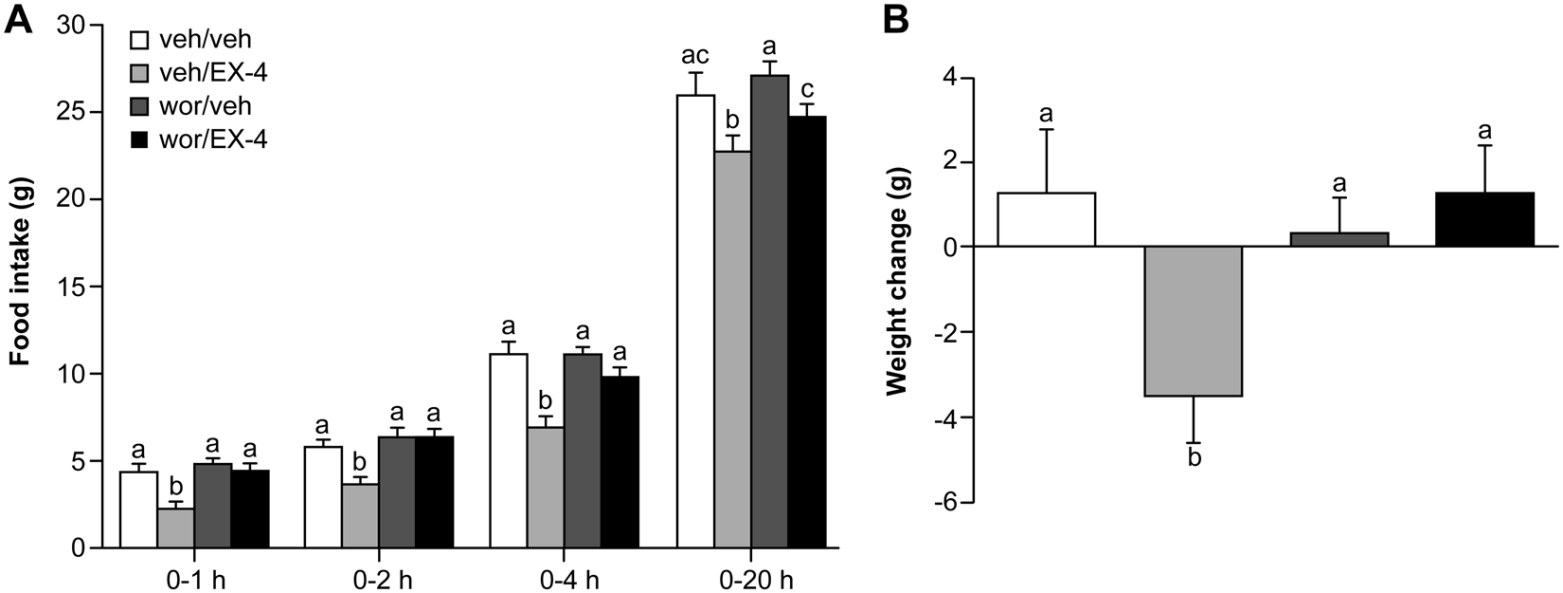
Food intake at 1, 2, 4, and 20 h (**A**) and 24 h body weight change (**B**) after ICV administration of wortmannin or vehicle and intrapretoneal injection of EX-4 or vehicle. A, Food intake was significantly reduced by EX-4, and 1.5 ng wortmannin reversed this reduction. B, Body weight was reduced significantly by EX-4, and this effect was attenuated by 1.5 ng wortmannin. Data are represented as mean ± SEM (N = 8 for each group). In each panel, groups that share the same letter are not significantly different from each other after obtaining a significant main effect by three-way ANOVA (**A**) and two-way ANOVA (**B**).

To examine the involvement of PI3K signaling in the food intake altering effects of EX-4, 1.5 ng wortmannin was administered via the ICV route prior to IP injection of EX-4 (3.2 µg/kg). Food intake was measured at 0–1, 0–2, 0–4, and 0–20 h after dark cycle initiation. Three-way repeated measures ANOVA (wortmannin × EX-4 × Time) revealed significant effects of wortmannin [F(1,44) = 10.8, *P* *<* 0.001], EX-4 [F(1,44) = 18.6, *P* *<* 0.001], Time [F(3,132) = 1913.9, *P* *<* 0.001], wortmannin × EX-4 [F(1, 44) = 4.4, *P* *<* 0.05] and EX-4 × Time [F(3, 132) = 4.7, *P* *<* 0.005] (Fig. 2A). The results indicated that peripheral EX-4 treatment combined with central vehicle (veh/EX-4) significantly reduced food intake as compared to the combinations of peripheral and central vehicle injections at each time point (veh/veh *vs*. veh/EX-4: 0–1 h, 4 ± 0.4 g *vs*. 2.4 ± 0.3 g, post-hoc *P* *<* 0.02; 0–2 h, 5.8 ± 0.4 g *vs*. 3.7 ± 0.3 g, post-hoc *P* *=* 0.013; 0–4 h,11.1 ± 0.7 g *vs*.7.0 ± 0.6 g, post-hoc *P* *<* 0.001; 0–20 h,26.0 ± 1.2 g *vs*. 22.7 ± 0.8 g, post-hoc *P* *<* 0.001). This reduction in food intake can be reversed by central 1.5 ng wortmannin injection prior to peripheral EX-4 treatment (wor/EX-4). Compared with the veh/EX-4 combination, wor/EX-4 resulted in significantly greater food intake (veh/EX-4 *vs*. wor/EX-4: 0–1 h, 2.4 ± 0.3 g *vs*.4.5 ± 0.3 g, *P* *<* 0.01; 0–2 h, 3.7 ± 0.3 g *vs*. 6.5 ± 0.4 g, *P* *<* 0.001; 0–4 h, 7.0 ± 0.6 g *vs*. 9.9 ± 0.5 g, *P* *<* 0.006; 0–20 h, 22.7 ± 0.8 g *vs*. 24.7 ± 0.7 g, *P* *<* 0.02]. Notably, there were no significant differences in food intake between the veh/veh and wor/EX-4 groups at any time point. Thus, EX-4 significantly decreased food intake, while wortmannin significantly attenuated the suppressive action of EX-4 on food intake at 0–1, 0–2, 0–4, and 0–20 h after 4 hours of fasting. Additionally, body weight was measured 24 hours after injections in these four groups. Two-way ANOVA (wortmannin × EX-4) revealed a significant effect of wortmannin × EX-4 [F(1,44) = 6.15, *P* *=* 0.017]. The wortmannin [F(1, 44) = 2.59, *P* *=* 0.114] or the EX-4 effect [F(1, 44) = 2.70, *P* *=* 0.108] was not significant. EX-4 treatment significantly reduced body weight (veh/EX-4 *vs*. veh/veh: −3.5 ± 1.1 g *vs*. veh/veh1.3 ± 1.5 g, post-hoc *P* *<* 0.005), and such an effect was attenuated by wortmannin (wor/EX-4 *vs*. veh/EX-4: 1.25 ± 1.1 g *vs*. −3.5 ± 1.1 g, post-hoc *P* *<* 0.005) (Fig. 2B).

### The effect of EX-4 on PI3K/AKT was inhibited by wortmannin

**Figure 3:**
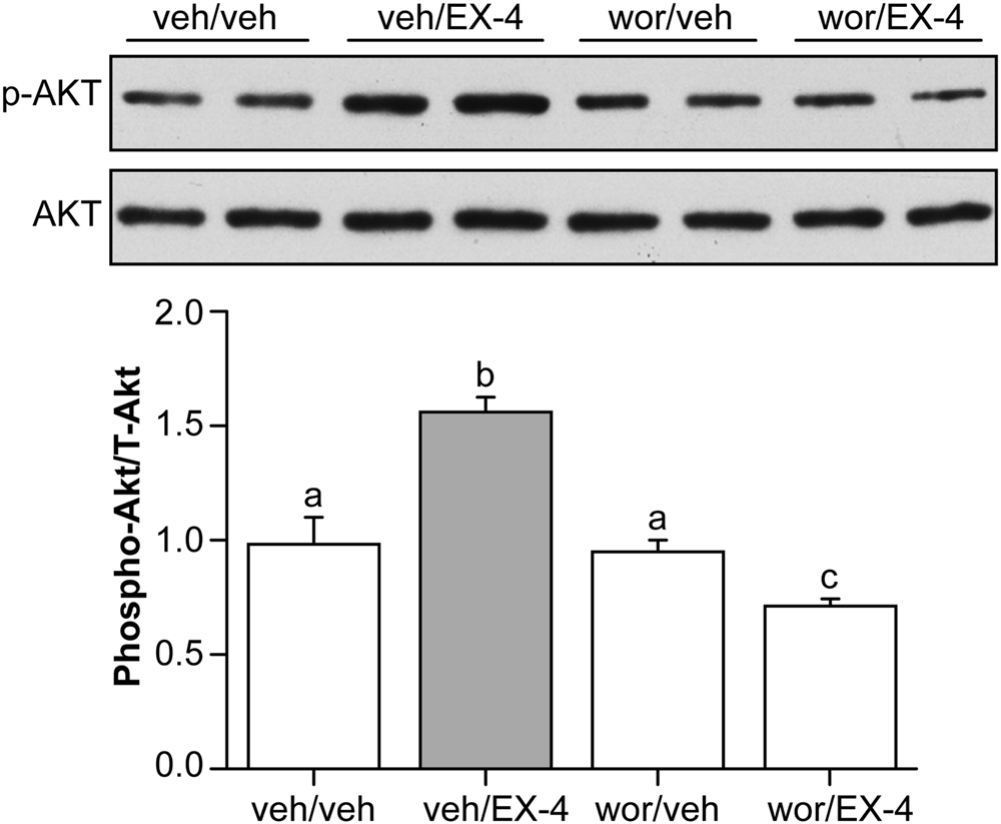
Activity of PI3K/AKT in the arcuate nucleus. EX-4 robustly increased phosphorylation of AKT in the ARC, and wortmannin inhibited this increase. In each panel, groups that share the same letter are not significantly different from each other after obtaining a significant main effect by two-way ANOVA. veh/veh: vehicle (5% DMSO in aCSF) third ICV and vehicle (water) IP; veh/EX-4: vehicle (5% DMSO in aCSF) third ICV and EX-4 IP; wor/veh: wortmannin third ICV and vehicle (water) IP; wor/EX-4: wortmannin third ICV and EX-4 IP.

Two-way ANOVA (Wortmannin × EX-4) revealed significant effects of wortmannin [F(1, 15) = 8.62, *P* *<* 0.01], EX-4 [F(1, 15) = 22.52, *P* *<* 0.001] and wortmannin × EX-4 [F(1,15) = 10.57, *P* *<* 0.007]. Third ICV wortmannin had no effect on AKT phosphorylation in ARC tissue at 3 h post-administration compared with central vehicle injection (wor/veh *vs*. veh/veh, *P* *=* 0.828). Intraperitoneal injection of EX-4 robustly increased phosphorylation of AKT 1 h post-administration compared with vehicle peripheral injection (veh/EX-4 *vs*. veh/veh, *P* *<* 0.001). This Ex-4 caused increase in AKT phosphorylation was inhibited by replacing the central vehicle with wortmannin (wor/EX-4 *vs*. veh/EX-4, *P* *<* 0.001). Together, these results indicate that the activity of PI3K/AKT was increased after peripheral injection of EX-4, and this change was reversed by a PI3K inhibitor (Fig. 3).

### The activity of IRS-1 was increased after EX-4 peripheral injection

**Figure 4:**
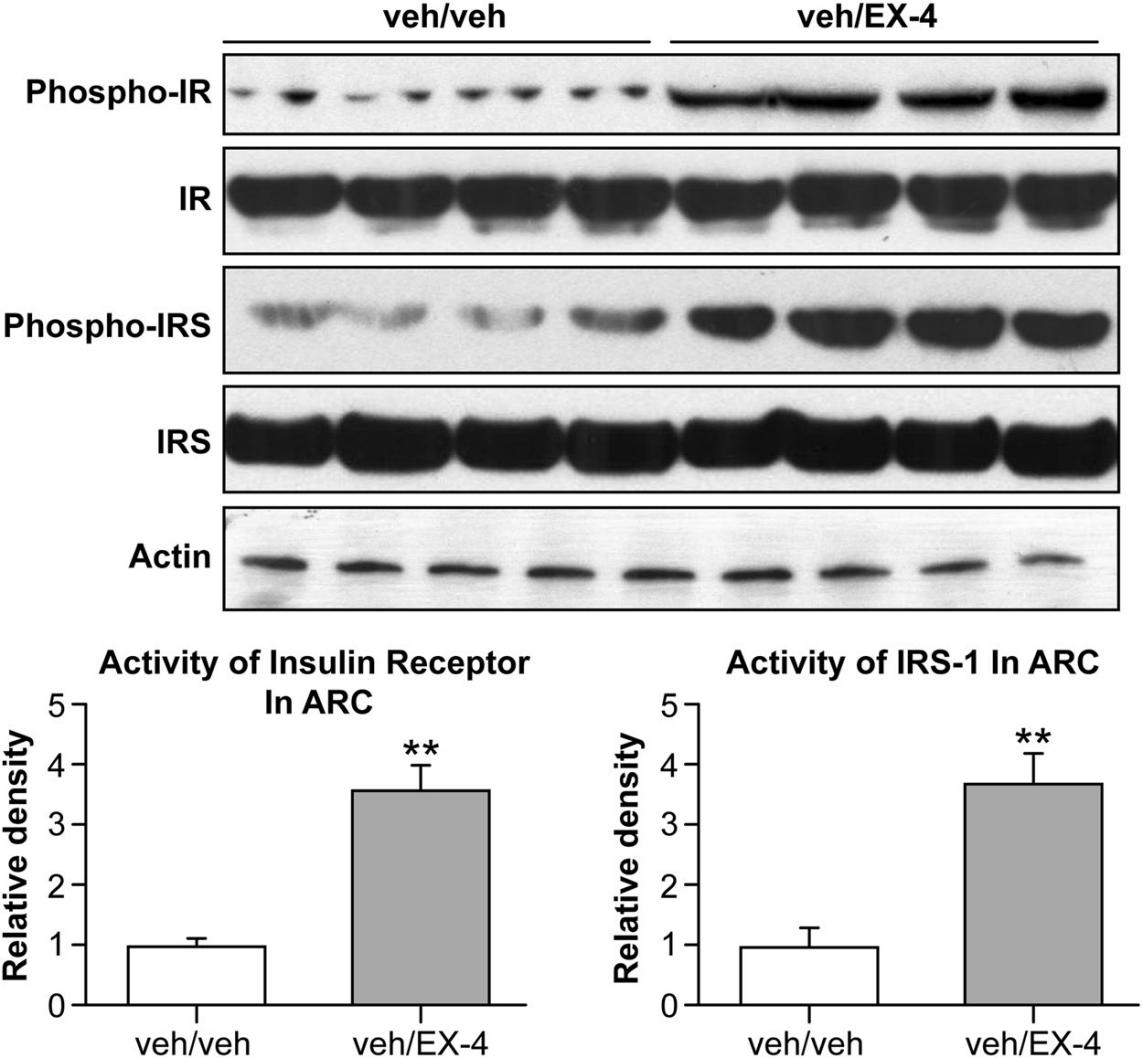
Activity of β subunit of the insulin receptor and IRS-1 in the ARC. EX-4 significantly activated insulin receptor and IRS-1 expression in the ARC. veh/veh: vehicle (5% DMSO in aCSF) third ICV and vehicle (water) IP; veh/EX-4: vehicle (5% DMSO in aCSF) third ICV and EX-4 IP. ** *vs*.veh, *P* < 0.01.

To test the role of insulin signaling in the activation of PI3K/AKT in the ARC by peripheral injection of EX-4, protein expression of the β-subunit of the insulin receptor and IRS-1 were determined by Western blotting. ARC samples were from the veh/veh and veh/EX-4 groups. Both the insulin receptor and IRS-1 were activated significantly, evidenced by an increase in protein levels (veh/EX-4, *P* *<* 0.05). These findings indicated that peripheral EX-4 injection might enhance ARC insulin signaling via insulin receptor signaling to PI3K/AKT (Fig. 4).

## Discussion

A previous study showed that EX-4 affects food intake and energy expenditure by activating GLP-1R^27^. In the present study, we found that EX-4, at a dose that induced anorexia, activated PI3K/AKT in the ARC. Furthermore, after injecting wortmannin into the third ventricle to block PI3K/AKT signaling^26^, the effects of EX-4 on food intake and body weight were significantly inhibited. Our results revealed that an insulin-IRS-1-PI3K/AKT-dependent pathway is involved in the mechanism through which GLP-1R in the ARC mediates food intake suppression.

PI3K encompasses a family of enzymes involved in cellular functions, such as cell growth, proliferation, differentiation, motility, and intracellular trafficking^28^. An essential downstream target of PI3K is AKT, which is activated via serine and threonine phosphorylation^29^. Current data suggest that hypothalamic PI3K signaling is important in the regulation of both energy balance and glucose metabolism^23, 30^. It has also been reported that PI3K increases the gene expression of *POMC* in the hypothalamic ARC^25, 31, 32^. Our previous study found that treatment with EX-4 increased *POMC* and decreased *NPY* expression in the hypothalamus of rats^8^. Meanwhile, there is evidence showing that GLP-1 receptors exist in the *POMC/CART* neurons, but not in *NPY/AgRP* neurons^33^. Previous studies have shown that EX-4 increases the activity of PI3K in peripheral tissues, such as liver, muscle, and pancreas^34–36^. There is possibility that EX-4 also increases the activity of PI3K in the *POMC/CART* neurons, which lead to increased expression of *POMC*, and resulted in decreased food intake.

In the current study, we aimed to determine whether the PI3K/AKT pathway also mediates the actions of EX-4 in the hypothalamus. We found that EX-4, at a dose of 3.2 μg/kg, significantly increased the phosphorylation of AKT in the ARC one hour after EX-4 administration, and at the same time food intake was notably decreased. If the phosphorylation of AKT is mediated by the PI3K signaling pathway, the increased phosphorylation of AKT induced by EX-4 should be reduced by pharmacological inhibition of the PI3K signaling pathway. Consistent with this hypothesis, injection of the PI3K inhibitor, wortmannin, into the third ventricle of the brain notably reduced the phosphorylation of AKT induced by EX-4, and the ability of EX-4 to decrease food intake was blocked. These data suggested that PI3K signaling mediates the downstream actions of EX-4 in ARC neurons. The study by Rupprecht *et al*.^14^ also suggested that PI3K/AKT pathway is involved in mediating suppressive effect of GLP-1R agonists on food intake, however, it was reported in their study that phosphorylation of AKT was suppressed when GLP-1 receptor was activated. One possible explanation for the discrepancy between our results is the different way of EX-4 injection (intraperitoneally vs. ICV). Furthermore, we focused on different region of the brain (ARC vs. hindbrain), and PI3K inhibitor was applied via 3^rd^ ICV in the current study, but via 4^th^ ICV in their study.

IRS-1 is an important component of insulin signaling, which is located upstream of PI3K/AKT^20, 21^. The activity of IRS-1 is increased after insulin binds with its receptor, which subsequently increases the activity of PI3K/AKT^20, 21^. It has also been reported that GLP-1 increased IRβ and IRS-1 in adipocytes^37^. To determine whether insulin signaling is involved in the activation of PI3K/AKT by EX-4, we assessed the activity of IRS-1 after peripheral EX-4 injection. Our results showed that phosphorylation of IRS-1 was markedly increased in ARC neurons one hour after EX-4 administration. These results provide evidence that EX-4 has a positive effect on IRS-1/PI3K/AKT signaling pathway in the ARC.

Schechter *et al*. previously described the production and secretion of insulin by fetal neurons in culture and demonstrated that neuronal synthesized insulin promoted neurofilamant distribution and axonal growth^38^. At the same time, exogenous insulin promoted neural differentiation and growth beyond central insulin^39^. The detailed molecular mechanisms by which EX-4 promotes insulin signaling in the ARC are still unclear. A possible explanation for the positive effect of EX-4 on IRS-1/PI3K/AKT signaling is that EX-4 promotes insulin secretion from neuronal cells or enhances insulin translocation through the blood-brain-barrier, which leads to higher brain insulin levels, and stronger activation of insulin signaling. However, there is also the possibility that EX-4 increases the activity of IRS-1 directly without the involvement of insulin. Further research is required to elucidate the full effects of EX-4 on IRS-1/PI3K/AKT signaling.

In summary, this study demonstrates a critical role of the PI3K/AKT pathway in regulating food intake in the ARC of rats and suggests that the effect of EX-4 in suppressing food intake is most likely due to its positive effect on IRS-1/PI3K/AKT signaling in the ARC.

## Methods

### Animals

Forty-eight adult male Sprague-Dawley rats (Harlan Industries, Frederick, MD) weighing 250–275 grams were individually housed under standard conditions (12:12 light/dark cycle, lights on at 00:00 h, 50–60% humidity). First, 16 rats were used for identifying the dose of ICV wortmannin that produced minimal effects on food intake. The remaining 32 rats were used for feeding tests. Rats were presented ad libitum access to standard rat chow (Teklad Global 18% Protein Rodent Diet, Harlan, Harlan Laboratories) and tap water except where noted. All animal procedures were approved by the Institutional Animal Care and Use Committee at Johns Hopkins University and conformed to the guidelines of the National Institutes of Health.

### Drugs

EX-4 (7000921, Bachem Americas, Inc. Torrance, CA) was dissolved in sterile water for injections, and a dose of 3.2 μg/kg was chosen based on our previous study^40^. Wortmannin (W1628, Sigma Aldrich, Inc. Saint Louis, MO) was dissolved in 5% DMSO in artificial cerebrospinal fluid (aCSF) (3525, R&D system, Minneapolis, MN) for ICV injections.

### Surgical procedures

At least one week after arrival, rats were anesthetized using a mixture of ketamine (90 mg/kg) and xylazine (2.7 mg/kg) and placed in a stereotaxic device (Kopf Instruments). A 23-gauge sterile guide cannula (Plastics One) was then implanted into the third ventricle (stereotaxic coordinates: −3 mm relative to the bregma, 0 mm lateral from the midline, and −8 mm ventral from the skull surface). Cannulae were attached to the skull by dental acrylic and jeweler’s screws. After 7 days of recovery, a third ventricle cannula was placed and verified by 30-minute water intake in response to administration of 10 ng (in 2 µl) angiotensin II. Only data from rats that drank at least 5 ml more water in response to angiotensin II than to vehicle (2 µl aCSF) were included in the analyses.

### Intracerebroventricular delivery of drugs and food intake

The purpose of this experiment was to determine whether ICV administration of wortmannin could block or attenuate the food intake suppressive effect of IP EX-4 injection. Sixteen rats were randomly assigned to 4 groups, and named as group “veh”, “0.5 ng”, “1.5 ng”, “5 ng”, respectively. After 7 days of recovery following cannulation surgery, food was taken away 4 hours before dark cycle onset. Two hours before dark cycle onset, three doses of wortmannin, 0.5, 1.5, 5 ng (in 2 µl/injection) or vehicle, were delivered to the third ventricle with a micropump (StoeltingCo., Wood Dale, IL) over a 2-minute period. Food was returned at dark cycle onset, and food intake was measured at 1, 2, 4, and 20 hours post-food presentation. Three experimental doses of wortmannin were chosen based on previous reports^27–29^. Based on the results of this test, we selected a dose of 1.5 ng wortmannin to test its effect on EX-4-associated food intake reduction.

Similar procedures were used to examine the effects of 1.5 ng wortmannin on EX-4-induced food intake reduction. Thirty-two rats were randomly assigned to 4 groups, with 8 rats in each group, and named as group “veh/veh”, “veh/EX-4”, “wor/veh”, and “wor/EX-4”. On the day of drug administration, food was taken away 4 hours before dark cycle onset. Two hours prior to dark cycle onset, rats received ICV injections of the PI3K inhibitor wortmannin (2 μl, 1.5 ng) or vehicle (2 μl 5% DMSO in aCSF). Five minutes prior to dark cycle onset, rats were injected with EX-4 intraperitoneally at a dose of 3.2 μg/kg or vehicle (water at a volume of 1 ml/kg). When the dark cycle began, rats were provided with standard chow, and measurements of food intake were taken 1, 2, 4, and 20 hours post-food presentation.

### Analysis of the protein levels of the β subunit of the insulin receptor and IRS-1 in the ARC by Western blotting

Seven days after the feeding test, PI3K signaling was examined after wortmannin and EX-4 administration. Food intake in response to wortmannin and EX-4 were measured using the previously described procedures. One hour after dark cycle onset, rats were sacrificed with rapid decapitation, and the brains were collected and flash frozen on dry ice and stored in −80 °C freezer until processing. Coronal hypothalamic brain sections (500 µm thick) were cut with a cryostat. Hypothalamic nuclei punches were then taken using a blunt 19-G needle. Punches for ARC were taken at −3.20mm relative to the bregma, and the tissue was immediately homogenized on ice in 50 µl tissue lysis buffer (Cell Lysis Reagent, Sigma Aldrich, St. Louis, MO) supplemented with protease and phosphatase inhibitor cocktails (Sigma-Aldrich). After 1 hour, the homogenates were centrifuged (15 min, 12,000 rpm, 4 °C), and supernatants were retained for protein determination. A protein assay kit (Pierce Microplate BCA, Thermo Fisher Scientific Inc., Rockford, IL) was used to determine the protein content. Whole tissue (13.7–26.1 μg) extracts were separated on 10% Bis-Tris SDS-PAGE gels (Life Technologies, Carlsbad, CA), followed by electrophoretic transfer to polyvinylidene fluoride membranes. Membranes were incubated with primary antibodies overnight at 4 °C and with secondary antibodies, and then they were conjugated to alkaline phosphatase at room temperature for 1 hour. Imaging was performed using a Typhoon FLA9500 imager (GE Healthcare), and densitometry was analyzed using Image J software. Primary antibodies for AKT, the phosphorylated site of Ser473 on AKT, IR, the phosphorylated site of Tyr1345 on IR, IRS-1, the phosphorylated site of Ser307 on IRS-1, and the secondary antibodies were purchased from Cell Signaling Technology (Danvers, MA, USA). The primary antibody against β-actin was purchased from Santa Cruz (Santa Cruz, CA, USA).

### Statistical analysis

All data are presented as mean ± SEM. All behavioral parameters were analyzed by two-way or three-way repeated measures ANOVA followed by a post-hoc Fisher LSD test as appropriate or Student *t* test where only two conditions were compared. Data from Western blots were analyzed by two-way ANOVA. All statistical analyses were conducted using Statistica (version 7.1, Tulsa, OK). Differences were considered significant at *P* *≤* 0.05.
